# Circular RNAs as potential biomarkers for male severe sepsis

**DOI:** 10.1515/biol-2022-0900

**Published:** 2024-07-24

**Authors:** Liang Jun, Zhonghua Wang, Shouhong Wang, Xiaolong Liao, Tiehe Qin, Weixin Guo

**Affiliations:** Department of Intensive Care, Guangdong Geriatrics Institute, Guangdong Provincial People’s Hospital, Guangdong Academy of Medical Sciences, Guangzhou 510080, China; Department of Intensive Care, Guangdong Geriatrics Institute, Guangdong Provincial People’s Hospital, Guangdong Academy of Medical Sciences, No. 106 Zhongshan Road, Guangzhou 510080, China

**Keywords:** circular RNAs, severe sepsis, circRNA array, microRNAs, transcriptome

## Abstract

Circular RNAs (circRNAs) play important roles in many human diseases. However, their role in the development of severe sepsis, a condition that remains one of the main causes of death in intensive care units, has not yet been defined. In this study, we interrogated the molecular mechanisms of circRNAs in severe sepsis. We profiled the expression levels of 5,680 circRNAs in plasma extracted from blood samples of 9 severe sepsis cases or 9 controls (male, age 78 ± 7) using the Human circRNA Array. To enrich protein-coding genes hosting severe sepsis-related circRNAs, we conducted gene ontology and pathways analyses. Out of the identified 760 differentially expressed circRNAs, 404 were upregulated while 356 were downregulated (fold change [FC] ≥2 or ≤−2, and false discovery ratio <0.05). Circ-0008285 (located in exons of *CDYL*), showed significant upregulation in severe sepsis with an FC of 13.7, and Bonferroni-corrected *P* < 0.05/5. In silico analysis identified Circ-0008285 interacting microRNAs as well as protein-coding genes. We systematically investigated the differential expression pattern of circRNAs in severe sepsis. The circRNAs we identified might serve as potential biomarkers for diagnosis and prognosis of sepsis.

## 1 Introduction

Sepsis is a life-threatening complication caused by the body’s extreme immune response to an infection [1]. Severe sepsis is triggered by infection combined with organ failure and hemodynamic instability. Severe sepsis occurs when there are incidences of organ failure, and remains one of the main causes of death in intensive care units [2,3]. Furthermore, a combination of severe sepsis and extreme hypotension leads to septic shock. Recently, genetic studies identify genetic loci and candidate genes associated with sepsis susceptibility. For example, rs5743708 in *TLR2*, rs1800629 in *TNFα*, and rs2569190 in *CD14* increase susceptibility to sepsis or septic shock [4,5,6].

Accumulating evidence suggests that severe sepsis induces changes in many protein-coding genes [7,8] or non-coding genes [9] in human genome, such as circular RNAs (circRNAs). circRNAs are considered single-stranded RNA molecules whose 5′ and 3′ ends are covalently closed as “backsplice” to form a continuous loop [10,11,12]. Whereas circRNAs were at first identified in the cytoplasm [13], thousands of circRNAs have been shown to be expressed in many types of human cells and tissues [10,11]. Most of the circRNAs come from the exons of protein-coding genes, while relatively small proportion come from either introns or intergenic regions [14,15,16,17,18]. The circular nature of the circRNA makes them notably stable even in the presence of RNase. Accumulating evidence suggests that circRNAs play pivotal roles in many human diseases, including sepsis. For instance, Ng et al. showed that RasGEF1B could regulate the stability of mature ICAM-1 mRNAs, thus fine-tuning the immune response and protecting host cells against infection, suggesting that circRNAs play a critical role in fine-tuning immune responses [19]. An abnormal immune system is one of the crucial triggers of sepsis. Therefore, circRNAs may potentially contribute to the effective treatment of sepsis. At present, an increasing number of circRNAs have been identified as potential markers in the diagnosis and treatment of sepsis [20], such as circRNA_0075723 [21], circRNA circFADS2 [22], and exosomal hsa_circRNA_104484 and hsa_circRNA_104670 [23].

To better understand the roles of circRNAs in the pathophysiology of severe sepsis, we profiled the expression levels of 5,680 circRNAs in 9 severe sepsis cases or 9 controls using high-throughput circRNA arrays and identified differentially expressed circRNAs. Then, gene ontology (GO) and pathway analyses were performed to identify novel pathways mediating the actions of circRNAs in sepsis. In addition, we created gene interaction networks using computational tools.

## 2 Methods

### 2.1 Clinical samples

Nine severe sepsis male patients and nine age-matched controls were used initial set. The patients’ blood samples were extracted within 24 h following the diagnosis of severe sepsis. The selected patients all meet the criteria of the 2016 guidelines for severe sepsis and are over 65 years old, while excluding patients with tumors or rheumatic diseases [2]. Three blood samples were merged randomly into one testing sample. Six circRNA arrays, i.e., three for the patient sample and three for the controls were performed. For the verification of differential circRNAs, 30 severe sepsis cases and 20 controls were used to validate the highly differentially expressed circRNAs by quantitative real-time PCR (qPCR).


**Informed consent:** Informed consent has been obtained from all individuals included in this study.
**Ethical approval:** The research related to human use has been complied with all the relevant national regulations, institutional policies, and in accordance with the tenets of the Helsinki Declaration and has been approved by the Clinical Research Ethics Committee of the Guangdong General Hospital.

### 2.2 Plasma collection

EDTA anticoagulant tubes were used to collect blood samples. The samples were then centrifuged in an Eppendorf centrifuge at 4°C and 3,000 rpm for 15 min. The plasma was separated into 1.5 mL RNA-free EP tubes and centrifuged at 13,000 rpm for 30 min to remove cell fragments. The collected plasma was transferred into new 1.5 mL RNA-free EP tubes and stored in −80°C liquid nitrogen.

### 2.3 Expression of circRNA and data normalization

We analyzed the expression of 5,396 human circRNAs by Arraystar Human circRNA Array (Kangcheng Inc). Total RNA from each of the samples was quantified using a NanoDrop ND-1000 at OD260 while the quality of the RNA was assessed by electrophoresis on a denaturing agarose gel. The sample preparation and microarray hybridization were performed following Arraystar’s standard protocols. Briefly, the total RNAs were digested with Rnase R (Epicentre, Inc.) to remove linear RNAs and enrich circRNAs. Then, the enriched circRNAs were amplified and transcribed into fluorescent cRNA (Arraystar Super RNA Labeling Kit; Arraystar). The labelled circRNAs were hybridized onto the Arraystar Human circRNA Arrays (8 × 15K, Arraystar) and incubated for 17 h at 65°C in an Agilent Hybridization Oven. The slides were washed and, then, the arrays were scanned by the Agilent Scanner G2505C. Raw data were extracted and analyzed using the Agilent Feature Extraction software (version 11.0.1.1). We then normalized (Quantile) the data using GeneSpring software. Thereafter, low-intensity filtering was performed, and the circRNAs that had flags in “P” or “M” (“All Targets Value”) in at least 1 sample were retained for further analyses.

### 2.4 Identification of differentially expressed circRNAs

Student *T*-test was used to identify differentially expressed circRNAs from severe sepsis cases versus controls. The false discovery ratio (FDR) was calculated by the Benjamini–Hochberg method [24]. The differentially expressed circRNAs with FDR < 0.05 were selected as significant. Hierarchical clustering [25] was performed to show the expression pattern of significant circRNAs.

### 2.5 qPCR

qPCR was used to validate the expression levels of differentially expressed circRNAs. The specific primers designed for backsplice junctions of each circRNA (divergent primer) are listed in Table S1. The GAPDH gene was used as an internal control. The Ct (cycle threshold, the number of cycles required for the fluorescent signals to cross the threshold of qPCR) value of each circRNA in each sample was measured. The expression levels of circRNAs were calculated as 2^−ΔCt^. △Ct was defined as Ct_circRNA_ – Ct_Inter___ctrl_. Student *T*-test was used to test the differential expression levels of circRNAs. Bonferroni-corrected *P* < 0.05 was considered significant.

### 2.6 GO and pathway analysis

For the differentially expressed circRNAs, topGO software [26] was used to perform GO [27], while the Kyoto Encyclopedia of Genes and Genomes (KEGG) [28] was used for pathway enrichment analysis. Fisher’s exact tests were used to find any overlap between the differentially expressed circRNAs sets and the GO terms/KEGG pathways. Benjamini-corrected FDR was set at <0.05 for the numbers of GO terms and pathways.

### 2.7 Data access

We deposited circRNA expression data to NCBI Gene Expression Omnibus with accession number GSE244903.

### 2.8 Statistical analysis for sequencing data

Agilent Feature Extraction software was used for raw data extraction. Statistical analysis of raw data was performed using the *R* software package. *T*-test was used for the differential analysis of circRNA expression. circRNAs having fold changes (FC) >2 and *P*-values <0.05 are selected as the significantly differentially expressed.

## 3 Results

### 3.1 Clinical characteristics

All participants included in this study were male, aged between 65 and 90 years. The initial samples included nine severe sepsis cases (age, 75 ± 8 years) and nine controls (age, 80 ± 8). The validation samples included 30 cases (age, 75 ± 7 years) and 20 controls (age, 76 ± 6). The age difference was not as significant at *P* > 0.05. Acute physiologic assessment and chronic health evaluation scores for the sepsis samples ranged between 23 and 35 (Table S2).

### 3.2 Identification of differentially expressed circRNAs in relation to severe sepsis

**Figure 1:**
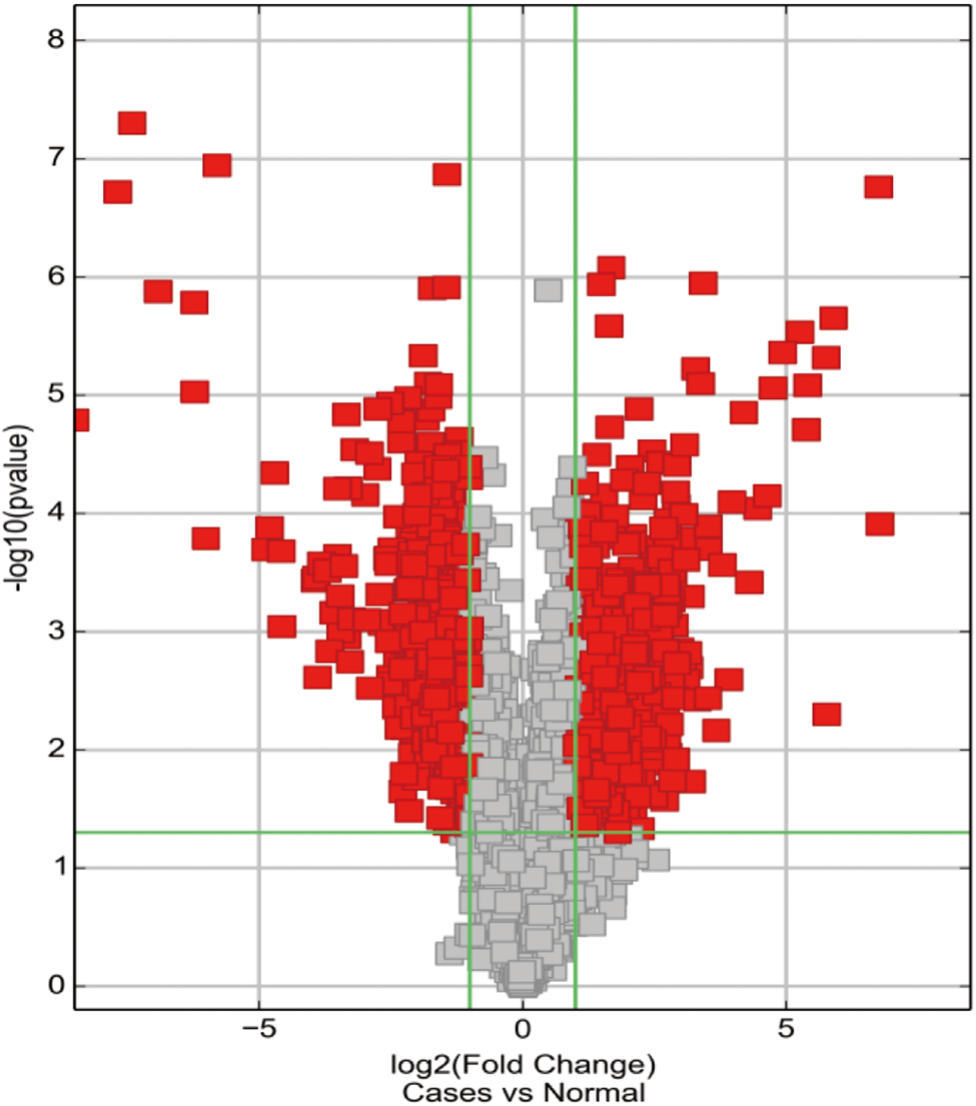
Volcano plot of differentially expressed circRNAs for severe sepsis. The *x*-axis showed the fold changes. The positive values indicated the upregulation and the negative values indicated the downregulation of genes in severe sepsis cases. The *y*-axis showed the log-10 transformed *P* values. The red rectangle indicated the differentially expressed circRNAs at *P* < 0.05. The vertical lines correspond to 2-fold change up and down respectively, and the horizontal line represented a *P* value of 0.05.

**Figure 2:**
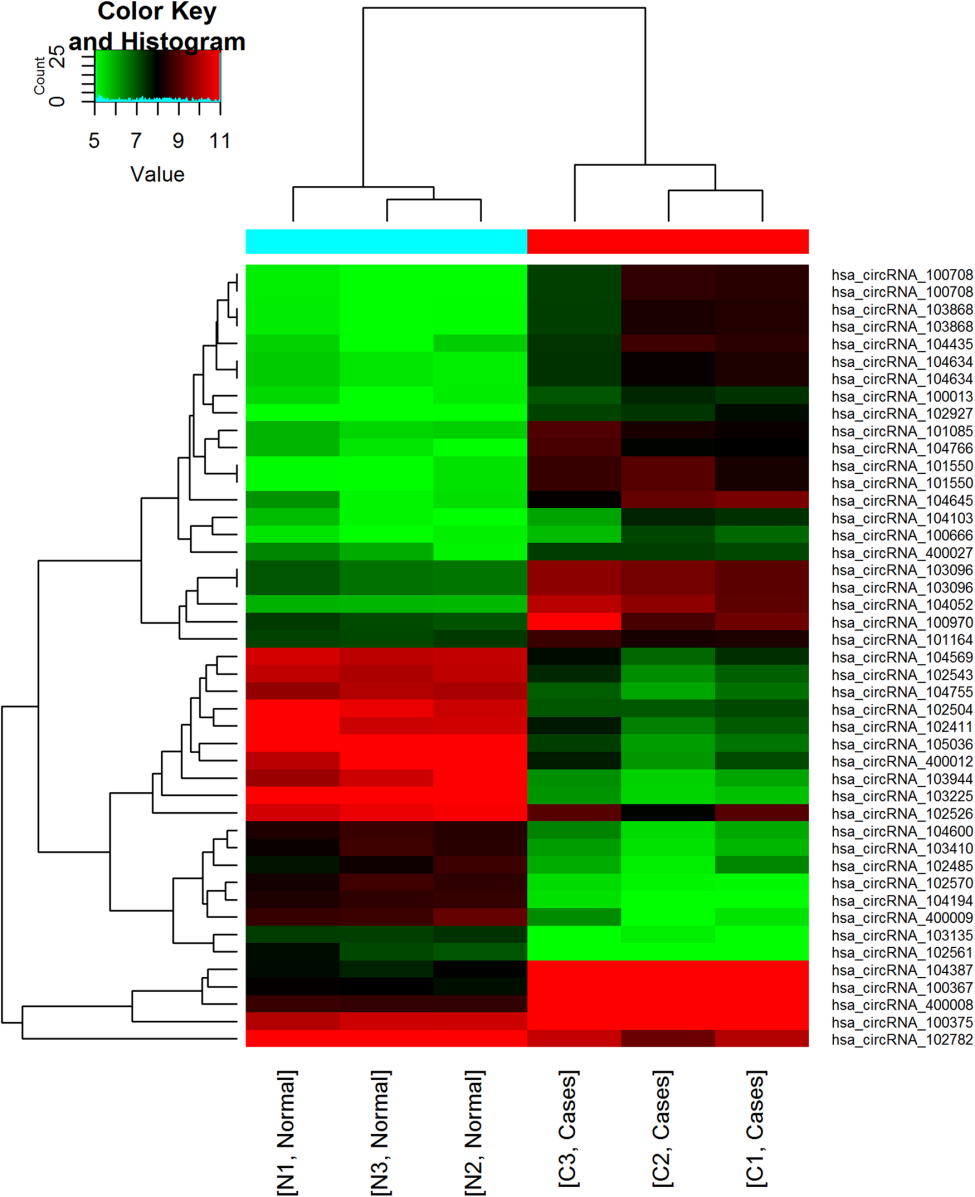
Heatmap of the top differentially expressed circRNAs for severe sepsis. Clustering analysis of differentially expressed circRNAs with 40 higher expression levels. circRNAs and samples were reordered by two-dimensional hierarchical clustering method, and the tree plots were the graphic representation of this process. Red color indicated upregulation and green color indicated downregulation.

There were 760 differentially expressed circRNAs in sepsis versus control cases at FC ≥ 2 or ≤ −2 and FDR < 0.05. Out of the 760, 404 circRNAs were upregulated while the rest were downregulated in severe sepsis (Figure 1). The upregulated circRNAs included 364 exonic, 20 intronic, 16 intragenic, and 4 antisense circRNAs. On the other hand, the downregulated circRNAs included 306 exotic, 33 intronic, 7 intragenic, and 10 antisense circRNAs. Figure 2 shows the expression pattern for the top 40 differentially expressed circRNAs.

### 3.3 GO and pathway enrichment analysis

To understand biological pathways underlying the differentially expressed circRNAs in severe sepsis, we performed GO and pathway enrichment analysis. The top enriched GO terms and pathways are shown in Table 1. At FDR < 0.05, the upregulated circRNAs were enriched for genes involved in many biological processes (GO-BP terms), such as intracellular transport, primary metabolic process, and immune systems process. Besides, there was an enrichment of upregulated circRNAs involved in pathways such as insulin signally pathway and B-cell receptor signaling. On the other hand, the downregulated circRNAs were enriched for genes involved in the regulation of gene expression and many other metabolic processes. The top GO-CC terms for the upregulated circRNAs were cytoplasm, intracellular organelle part, or intracellular, while the terms for the downregulated circRNAs were nucleus or intracellular. The top GO-MF terms for the upregulated circRNAs were protein binding, ployA RNA binding, or enzyme binding, while those for the downregulated circRNAs were DNA binding, nucleic acid binding, or nucleic acid binding transcription factor activity.

**Table 1:** Top GO terms and pathways for differentially expressed circRNAs in sepsis

GO term/pathway	Resource	Fold change	*P* value	FDR
**Upregulated circRNAs**				
Intracellular transport	GO-BP	1.45	1.72 × 10^−26^	3.50 × 10^−23^
Primary metabolic process	GO-BP	1.10	1.20 × 10^−20^	6.11 × 10^−18^
Immune system process	GO-BP	1.31	1.98 × 10^−20^	8.64 × 10^−18^
Cytoplasm	GO-CC	1.22	1.41 × 10^−80^	1.04 × 10^−77^
Intracellular organelle part	GO-CC	1.30	6.63 × 10^−79^	2.09 × 10^−76^
Intracellular	GO-CC	1.13	2.18 × 10^−74^	2.69 × 10^−72^
Protein binding	GO-MF	1.16	3.15 × 10^−49^	3.50 × 10^−46^
Poly(A) RNA binding	GO-MF	1.53	5.75 × 10^−23^	2.13 × 10^−20^
Enzyme binding	GO-MF	1.39	5.08 × 10^−19^	1.13 × 10^−16^
Lysosome	KEGG	11.29	5.13 × 10^−12^	1.51 × 10^−9^
Insulin signaling pathway	KEGG	8.17	6.77 × 10^−9^	9.95 × 10^−7^
Regulation of actin cytoskeleton	KEGG	5.67	2.13 × 10^−6^	8.97 × 10^−5^
B-cell receptor signaling pathway	KEGG	5.47	3.40 × 10^−6^	1.11 × 10^−4^
Endocytosis	KEGG	5.26	5.55 × 10^−6^	1.63 × 10^−4^
**Downregulated circRNAs**				
Regulation of gene expression	GO-BP	1.20	7.25 × 10^−13^	2.27 × 10^−9^
Regulation of nitrogen compound metabolic process	GO-BP	1.16	7.64 × 10^−10^	2.02 × 10^−7^
RNA metabolic process	GO-BP	1.16	7.95 × 10^−10^	2.02 × 10^−7^
Nucleus	GO-CC	1.11	1.11 × 10^−8^	8.18 × 10^−6^
Intracellular	GO-CC	1.03	2.92 × 10^−5^	1.08 × 10^−2^
DNA binding	GO-MF	1.28	3.83 × 10^−13^	4.26 × 10^−10^
Nucleic acid binding	GO-MF	1.17	9.64 × 10^−10^	5.37 × 10^−7^
Nucleic acid binding transcription factor activity	GO-MF	1.20	3.29 × 10^−4^	3.66 × 10^−2^

### 3.4 Validation of the top differentially expressed circRNAs

**Figure 3:**
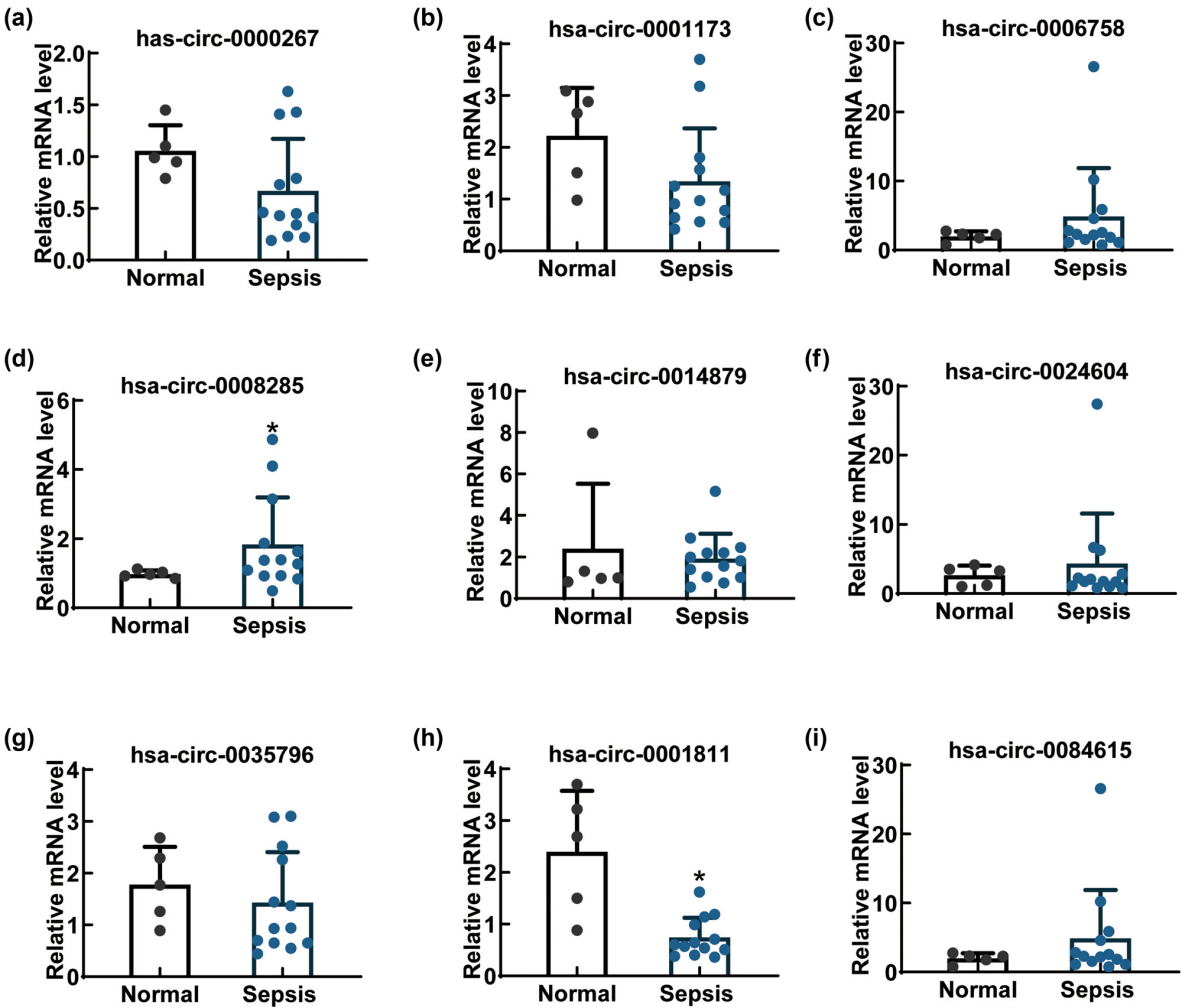
The expression levels of circRNAs in the replication samples. The expression levels of hsa-circ-0000267 (a), hsa-circ-0001173 (b), hsa-circ-0006758 (c), hsa-circ-0008285 (d), hsa-circ-0014879 (e), hsa-circ-0024604 (f), hsa-circ-0035796 (g), hsa-circ-0001811 (h), and hsa-circ-0084615 (i) were detected using qPCR assay. *P < 0.05.

Through reference review, we selected 9 (hsa-circ-0000267, hsa-circ-0001173, hsa-circ-0006758, hsa-circ-0008285, hsa-circ-0014879, hsa-circ-0024604, hsa-circ-0035796, hsa-circ-0001811, and hsa-circ-0084615) of the top 40 circRNAs for qPCR validation. As shown in Figure 3, the difference in circ-0008285 expression was significant.

### 3.5 Construction of the hsa-circ-0008285 gene interaction networks

**Figure 4:**
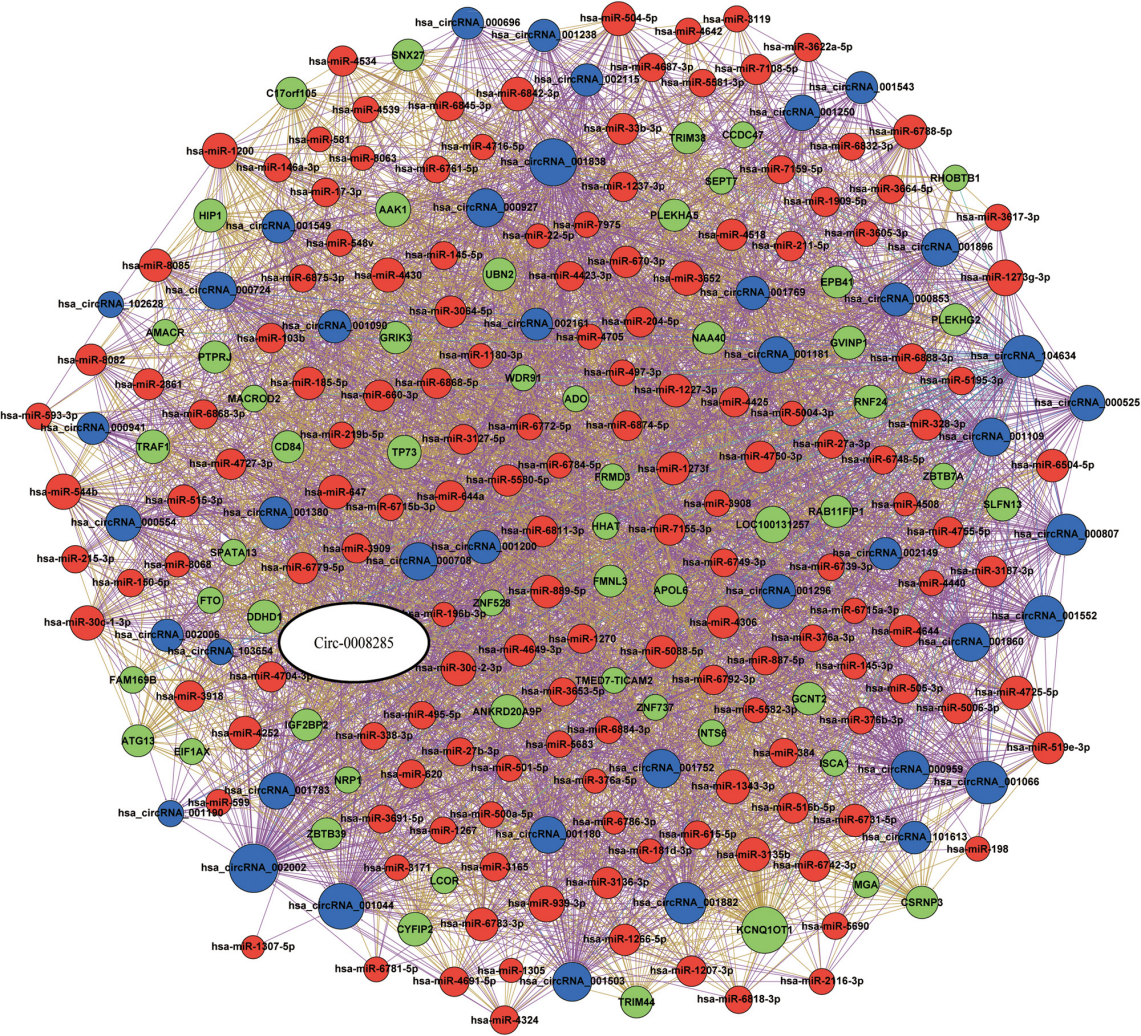
A hsa-circ-0008285-derived gene network. hsa-circ-0008285 targeted mRNAs (*n* = 60, green circular), circRNAs (*n* = 39, blue circular), and miRNAs (*n* = 117, red circular) were predicted by bioinformatics, luciferase reporting system, CHIRP, and RNA sequencing.

To better understand the role of hsa-circ-0008285, we used computational tools to predict hsa-circ-0008285-interacting mRNAs, miRNAs, or other circRNAs. From the same sample as the circRNA chip, we carried out whole transcriptome detection of mRNA, micro-RNA as well as mRNA related to circ-0008285 as shown in Figure 4. There were a total of 117 miRNAs, 60 mRNA, and 39 circ-RNAs.

## 4 Discussion

circRNAs are in low abundance but notably stable and widespread in human tissues and cells [29,30]. Recently, thousands of circRNAs have been identified in various tissues and organs, and most of the circRNAs were associated with many diseases [30,31,32,33,34].

In this study, we systematically analyzed shifts in the expression of 5,680 circRNAs in severe sepsis using high-throughput circRNA arrays. The analysis identified 760 differentially expressed circRNAs. The differentially expressed circRNAs suggested many novel biological processes or pathways that are involved in the pathophysiology of severe sepsis. We have, for the first time, systematically identified circRNAs on a genome-wide scale for severe sepsis. We identified hundreds of circRNAs that were differentially expressed in severe sepsis. According to the qPCR results, we validated circRNAs such as hsa-circ-0008285 (exons of *CDYL*) and hsa-circ-0001811 (exons of *STAU2*) as potential biomarkers for severe sepsis.

In recent studies, hsa-circ-0008285 has been shown to adsorb Let-7c and inhibit the entry of interferon regulatory factor 4 into the nucleus, thereby promoting the expression of C/EBP-δ, promoting vascular inflammation, and inducing M1 polarization in macrophages [35]. Although hsa-circ-0008285 has been confirmed to be involved in inflammation, its expression and role in severe sepsis have not been reported. In addition, hsa_circ_0001811 was low expressed in gastric cancer [36], and targeted the miR-589/CAPZA1 axis to inhibit the progression of gastric cancer [37]. However, most of the circRNAs are still less studied in severe sepsis. More comprehensive functional studies in the future are needed to validate their roles and molecular mechanisms in severe sepsis.

GO and pathway analysis studies showed that sepsis-related circRNAs were localized in exons of protein-coding genes. These circRNAs were enriched for genes involved in many metabolic processes or immune functions. These results supported the pathology of sepsis as a systemic inflammation in response to infection. circRNAs may interact with their hosting genes or miRNAs to regulate such processes or pathways.

However, this study only performed six high-throughput circRNA arrays. The small sample size reduced the confidence of the initial results defining the differentially expressed circRNAs. In addition, both the patients and control samples included in this study were male, which led to reduced reliability of the research results. We plan to conduct further follow-up validation studies for the top results to provide more reliable results.

## Supplementary Material

Supplementary Table
