# Supplementary material for: Circular RNAs as potential biomarkers for male severe sepsis
Source: Open Life Sci. 2024 Jul 24;19(1):20220900. doi: 10.1515/biol-2022-0900 (PMC11282911; doi:10.1515/biol-2022-0900)
Supplement: Supplementary Table [file biol-2022-0900-sm.pdf]

Supplementary material

Table S1: Primers of the circRNAs

GAPDH-F	GAGTCAACGGATTTGGTCGT
GAPDH-R	GACAAGCTTCCCGTTCTCAG
hsa_circ_0000267 F	CAGGCATTCCCAGATGCTG
hsa_circ_0000267 R	GTCGGTGTTAAAAGGCGGC
hsa_circ_0001173 F	GCTGGCAATTCAAACACACA
hsa_circ_0001173 R	CTACGGGAGGAGAACAAGCA
hsa_circ_0006758 F	TTCTGCTCCGAGGTAAGGAC
hsa_circ_0006758 R	GACTTTGTCTCCCATTCCCG
hsa_circ_0008285 F	CCACCGAACCAAATACTCTGTC
hsa_circ_0008285 R	GGTCGGAGCTTTATTGGGC
hsa_circ_0014879 F	CCTTTGCTGGACATCTTGA
hsa_circ_0014879 R	CTCTCCCTGTACGTTCTTATC
hsa_circ_0024604 F	TGTTGGTGACTCTCGGTT
hsa_circ_0024604 R	AGTTGAGCGCATTGCATC
hsa_circ_0035796 F	TCGAGTAAATGAATGCTCC
hsa_circ_0035796 R	TAGGCACTGTTGGACACACA
hsa_circ_0001811 F	CCATGTCTGCTCACCAAGAC
hsa_circ_0001811 R	GGAGAGCCTGCCATCTACAG
hsa_circ_0084615-F	GCCTTTCCTCCCATTCTT
hsa_circ_0084615-R	TCAGAGGTGCTTCAAGGAA

Table S2: General clinical data of patients in the circRNA array

Clinical features		Case (*n*)
Gender	Male (*n*)	18
	Female (*n*)	0
Age (years)		77.8 ± 8.2
Status	Healthy	9
	Severe sepsis	9
APACHEII (For severe sepsis)		29.4 ± 2.7
